# Dynamic Stability of Tensegrity Structures—Part I: The Time-Independent External Load

**DOI:** 10.3390/ma16020580

**Published:** 2023-01-06

**Authors:** Paulina Obara, Justyna Tomasik

**Affiliations:** Faculty of Civil Engineering and Architecture, Kielce University of Technology, al. Tysiąclecia Państwa Polskiego 7, 25-314 Kielce, Poland

**Keywords:** tensegrity structures, initial prestress forces, infinitesimal mechanism, stiffness, frequency

## Abstract

The paper contains a parametric analysis of tensegrity structures subjected to time-independent external loads. A complete dynamic stability analysis is a three-step process. The first stage involves the identification of self-stress states and infinitesimal mechanisms. The next stage concentrates on the static and dynamic behavior of tensegrities under time-independent external loads, whereas the third is under periodic loads. In this paper, the first two stages are carried out. The structures built with the most popular tensegrity modules, Simplex and Quartex, are considered. The effect of the initial prestress on the static parameters and frequency is analyzed. To assess this behavior, a geometrically non-linear model is used.

## 1. Introduction

The paper concerns the static and dynamic behavior of tensegrity structures. Unlike conventional cable-strut frameworks, tensegrity structures are characterized by a system of internal forces, which keeps the elements of the structure in stable equilibrium (self-stress state). The most interesting for all are tensegrity structures characterized by the occurrence of infinitesimal mechanisms. In the absence of self-stress (initial prestress forces), such systems are unstable, i.e., geometrically variable. The stabilization occurs only after the introduction of initial prestresses. Their modification allows for controlling the static and dynamic parameters of the structure.

The subject of tensegrity systems is very popular. The main features of tensegrity structures are well-known. However, the topic of tensegrity systems is still being explored. The total number of papers on the Web of Science homepage using topic “Tensegrity” from 2001 to 2020 is approximately 1000 [1]. From the beginning of the idea of tensegrity, i.e., the 1960s, to the present day, the most popular subject of papers is the search for geometrical configuration (form-finding)—a few sample papers from the last two years [1,2,3,4]. Other popular applications can be divided into three main areas:

- algorithms changing the shape of the structure—optimization algorithms aimed at generating new topologies; the new topology aims to achieve the desired performance criteria, such as stiffness level [5,6,7,8],
- shape control methods—methods examining the change of the shape of the structure under the influence of external forces [7,9,10,11,12,13,14,15,16,17],
- parametric analysis taking into account the impact of the initial prestress on the static and dynamic behavior of structures [18,19,20,21,22,23,24,25,26,27].

The first area concerns the optimization of tensegrity structures. In [5], the influence of changing the position of the struts (control of the length of the cables) on the dynamic characteristics was investigated. The subject of the consideration was a tensegrity module consisting of six struts and 18 cables. Both the non-linear dynamic model and the linearized version were used to describe the behavior of the module. It was shown that, with the increase in the prestress level, the differences between both approaches decrease. In [6], the process of optimizing the prestressing of a cantilever flat tensegrity structure was presented. In turn, Bel Hadj Ali [7] carried out the optimization of a tensegrity footbridge built of six fifteen-strut pentagonal modules. Studying the dynamic efficiency of the footbridge, the author was looking for a cost-effective design solution. In [7], a method of designing the initial stresses using the eigenfrequencies of tensegrity structures was presented. The optimization of the self-stress state was carried out in conditions in which the tensegrity system can reach its maximum stiffness state. The analysis was carried out on the example of a flat structure built of three X modules and a three-dimensional structure, built with five Quartex modules. Caluwaerts and Carbajal [8] optimized the shape of a single tensegrity module—a regular polyhedron built of six struts and a tower built of three Quartex modules. The number of cables in these structures was determined in the optimization process.

The most important research on the analysis of tensegrity structures focuses mainly on the second area, i.e., on the control of the shape of the structure under the influence of external forces. The search for the force-displacement relationship can be carried out by examining the damping and frequency of vibrations and by examining the change in geometry. Control methods are divided into passive and active. For example, Skelton and his team [9] used a passive method and developed a nonlinear dynamic analytical model of tensegrity structures. In the proposed method, the kinematics is described by the position and velocity of the ends of the struts. The model was used for shape control and design of foldable structures. In another work [10], Skelton described further analytical models allowing for easier control of the shape of tensegrity structures. In turn, in [11], the behavior of pre-stressed tensegrity plates built with 36 and 256 Quartex modules was investigated. The considerations take into account the influence of the number of loose cables on the behavior of the plates. Fraternali, with a team [12], studied the behavior of a tower built of four three-strut modules (Simplex modules). Faroughi and Lee [13] analyzed the behavior of pre-stressed tensegrity plates composed of 20 and 36 Quartex modules. The authors investigated the change in the displacement of plates loaded with a harmonic force. They used a linear model in the analysis. In turn, in [14], a new spatial tensegrity element was proposed for modelling the nonlinear dynamics of trusses subjected to a time changing load. The application of the model was presented on the example of a pre-stressed Quartex module and a tensegrity plate made of 20 such modules. In [15], the authors used the Euler-Bernoulli beam element to analyze the dynamic behavior of planar and spatial tensegrity structures, taking into consideration the influence of the axial force on the transverse stiffness. A different model was used in [16]. Initially, the authors formulated the single-element member and then introduced a grouped multi-element member, which was used to develop a dynamic model to study the dynamic properties of tensegrity structures. In [17], a model using the dynamic relaxation method in combination with neural networks was proposed to calculate the nodal displacements of tensegrity structures. In [7], the authors investigated the influence of the self-stress state on the dynamic behavior of the structure.

Compared to the abundant literature on the above-mentioned areas, the parametric analysis, determining the influence of initial prestress on the static and dynamic properties of tensegrity structures, has been developed slightly. In [18], the influence of the initial prestress on the static properties of full-scale tensegrity skeleton of White Rhino was carried out. The static parametrical analysis of structures build with tensegrity modules, i.e., Simplex and Quartex, was performed, among others, in [19,20,21]. In turn, in [22,23,24], the influence of the initial prestress on the dynamic properties of the Simplex module was analyzed. In [25], the impact of the initial prestress on the dynamic properties of a six-strut spherical module and a two-module cylindrical tensegrity model was considered. Bel Hadj Ali and Smith [26] determined the effect of the self-stress level on the vibration frequency of a footbridge built of six fifteen-strut modules. However, the dynamic of tensegrity structures built with three- and four-strut modules were analyzed in [27].

The analysis of the literature shows that the vast majority of works concerns the tensegrity design, the search for stable forms, optimization algorithms, methods of controlling the shape of tensegrity structures under the influence of external loads and discusses the use of these structures. Against this background, parametric analysis evaluating the influence of the state of self-stress on the static and dynamic properties of tensegrity structures is the subject of a few studies. In addition, these works relate to specific solutions. No monographic studies concisely describe the behavior of the full spectrum of structures. The studies known to the authors lack the analysis of dynamic stability understood in terms of the Bolotin approach [28]. This problem is often confused with the issues of impulse loads. Dynamic stability analysis leads to the determination of parametric resonance areas (dynamic instability) that are dangerous for the durability of the structure. From the point of view of the physical interpretation of the phenomenon of dynamic instability, if the load parameters are within the defined limits of instability, the structure experiences vibrations with increasing amplitude. There is an abundant literature on parametric vibrations that essentially defines all the basic issues. Nevertheless, tensegrities are a special example of structures. They are characterized by an additional parameter, which is the self-stress state, which affects the shape and range of instability areas.

Taking the above into account, it seemed reasonable to take up the subject of dynamic analysis, in particular the analysis of dynamic stability of tensegrity structures. The main purpose of the work was to examine the behavior of tensegrity structures under the influence of periodic loads and to find the answer to the question of whether and to what extent initial prestress affect the distribution of areas of dynamic instability. The implementation of the formulated objective required:

- performing a qualitative assessment involving the identification of self-stress states and infinitesimal mechanisms,
- performing a quantitative assessment of the behavior of tensegrity structures under time-independent external loads:
  − determination of the minimum and maximum initial prestress levels,
  − assessment of the impact of initial prestress on the structural displacements,
  − assessment of the impact of initial prestress on the rigidity of structure,
  − assessment of the impact of initial prestress on the normal forces,
  − assessment of the impact of initial prestress on the effort of structure,
- performing parametric analysis determining the influence of initial prestress on dynamic properties, including the frequency and forms of vibrations,
- conducting a dynamic stability analysis leading to the determination of the resonance frequency of periodic extortions and instable areas as a function of initial prestress.

Due to the wide scope, the work was divided into two parts. The first part contains the identification of self-stress states and infinitesimal mechanisms, the analysis of the static and dynamic behavior of tensegrities under time-independent external loads, whereas the second part will contain a dynamic stability analysis under periodic loads. This study aims to describe the behavior of the full spectrum of tensegrity structures in order to indicate the general characteristics of tensegrity structures, allowing for a better understanding of the specificity of this type of structure.

First, to illustrate the behavior of structures characterized by self-stress states and infinitesimal mechanisms, the authors propose to consider the simplest truss consisting of two elements. Despite the fact that this structure is not a tensegrity, its behavior fully reflects the behavior of tensegrity structures and makes it possible to determine of impact of initial prestress level S on the static and dynamic parameters in explicit form contained in this study.

Next, tensegrity structures built with the modified Simplex and Quartex modules are considered. The aim of analysis was to compare three towers build with the most popular tensegrity modules. In this work, the authors tried to cover many different examples of tensegrity structures. The linear connection with various ways of joining the modules and different support conditions are considered. The Simplex modules could only be connected in one way, whereas the Quartex modules could be in two ways. In the first connection, the struts overlap in a plan view, while in the second, they form a star. The analysis was intended to fully describe the static and dynamic behavior of tensegrities by tracking the effort of the structures and checking the stability of the examined structures at each step of the analysis. The static behavior was studied by the analysis of the influence of initial prestress and external load on normal forces and on the rigidity of the structures. In the literature on tensegrity structures known to the authors, the influence of initial prestress level on stiffness was studied only as the effect on the displacements. In a structure with one non-zero displacement, the measure of stiffness depends only on this non-zero displacement. In this case, the assessment of the behavior of this displacement (local assessment) is also an assessment of the behavior of the entire structure (global assessment). In the case of structures with many degrees of freedom, it is not possible to trace all displacements for objective reasons. Therefore, it seems reasonable to introduce a parameter that will reliably determine the effect of the state of self-stress on the total rigidity of the structure at a given load. In the literature on tensegrity structures, however, no parameter characterizing the change in rigidity has been found. The authors propose a parameter to compare the rigidity of the structures. This parameter expresses the ratio of two strain energies, measured at the minimum and at the *i*-th level of initial prestress. It is the measure of changes in the stiffness caused by the influence of the initial prestress on the total stiffness of the structure at a given load. This parameter expresses the ratio of two strain energies, measured at the minimum and at the *i*-th level of initial prestress. It is the measure of changes in the stiffness caused by the influence of the initial prestress on the total stiffness of the structure at a given load. In turn, the dynamic behavior was studied by analyzing the influence of initial prestress and external load on frequencies.

A nonlinear analysis is used, assuming the hypothesis of large displacements. The presented study proves that some tensegrities are characterized by an abnormal dynamical behavior. These parametric considerations are crucial for the dynamic stability analysis of the behavior of tensegrity structures under periodic loads, which will be the subject of the second part of the paper. The dynamic stability analysis cannot be carried out without the analysis presented in this paper.

## 2. Methods of Analysis

The tensegrity structure is an n-element spatial truss (e=1, 2,…, n) with m  degrees of freedom described by a displacement vector $q\left(\in {\mathbb{R}}^{m\times 1}\right)$. The most characteristic feature of tensegrity structures is the self-stress state that stabilizes existing infinitesimal mechanisms. The modification of the level of self-stress state in tensegrity structures allows for controlling their dynamic properties. Another specific property of these systems is the size of the displacements, which can be large even with small deformations. To evaluate the static and dynamic behavior, a geometrically non-linear model is used, assuming the hypothesis of large displacements [20,21,29,30,31]. The non-linear theory of elasticity in terms of the Total Lagrangian (TL) was adopted as the basis for formulating tensegrity lattice equations. The non-linear equation of motion is as follow:

(1) $$M\ddot{q}\left(t\right)+\left[K_{L}+K_{G}\left(S)+K_{GN}(N\right)+K_{NL}\left(q\right)\right]q\left(t\right)=P$$

where $M\left(\in {\mathbb{R}}^{m\times m}\right)$ is a consequent matrix of masses, $\ddot{q}\left(\in {\mathbb{R}}^{m\times 1}\right)\;$ is an acceleration vector, $K_{L}\left(\in {\mathbb{R}}^{m\times m}\right)$ is a linear stiffness matrix that depends on an expansion matrix $B\left(\in {\mathbb{R}}^{n\times m}\right)$ and an elasticity matrix $E\left(\in {\mathbb{R}}^{n\times n}\right)$, $K_{G}\left(S\right)\left(\in {\mathbb{R}}^{m\times m}\right)$ is a geometric stiffness matrix that depends on an initial prestress S, $K_{GN}\left(N\right)\left(\in {\mathbb{R}}^{m\times m}\right)$ is a geometric stiffness matrix that depends on axial forces  N, which results from external loads, $K_{NL}\left(q\right)\left(\in {\mathbb{R}}^{m\times m}\right)$ is a non-linear displacement stiffness matrix and $P\left(\in {\mathbb{R}}^{m\times 1}\right)$ is an external load vector. In this part, the time-independent loads $P=P\left(t=0\right)$ are considered. The explicit matrices forms can be found, for example, in [20].

The first stage of the analysis leads to the identification of the immanent features, which are self-stress states and infinitesimal mechanisms. The existence of these features depends only on the expansion matrix $B\left(\in {\mathbb{R}}^{n\times m}\right)$, so the geometrical and mechanical characteristics do not affect them (the elasticity matrix is an identity matrix E=I). The self-stress state is considered as an eigenvector $y_{S}$ related to the zero eigenvalue of the compatibility matrix $BB^{T}\left(\in {\mathbb{R}}^{n\times n}\right)$, whereas the mechanism is an eigenvector $x_{S}$ related to the zero eigenvalue of the stiffness matrix $B^{T}IB\left(\in {\mathbb{R}}^{m\times m}\right)$ [18,19,21,22,32].

The second stage of analysis leads to the determination of the influence initial prestress levels on the static and dynamic behavior of structures. First, it is necessary to determine the range of initial pre-stresses, which is characteristic for each structure. The minimum prestress level $S_{min}$ is related to the appropriate distribution of normal forces in the elements of the structure. The external load can cause a different distribution of normal forces, and it can be corrected by the introduction of a proper level of initial prestress. The maximum prestress level $S_{max}$ is related to the load-bearing capacity of the most stressed elements.

### 2.1. Static Analysis

For traditional lattice structures, the static analysis can be performed assuming small displacements, i.e., a linear geometric model. It is an improper approach for the analysis of tensegrity structures. The quasi-linear model (second order theory) is also inadequate. Both approaches do not take into account the stiffening of the structure under the influence of external load. In tensegrity structures, the load causes displacements in accordance with the form of the infinitesimal mechanism that induces additional prestress of the structure—tensile forces generate additional tension in the cables and compression in the struts. For such regimes, the initial response should not be used to determine the behavior of the structure. Therefore, the analysis must be carried out with the assumption of the hypothesis of large displacements (third order theory).

To illustrate the influence of external loads on the stiffening, two approaches are used. The applied methods are the quasi-linear approach (second order theory):

(2) $$\left[K_{L}+K_{G}\left(S\right)\right]q=P,$$

and non-linear approach (third order theory):

(3) $$\left[K_{L}+K_{S}\right]q=P;\;\;\;\;\;\;K_{S}=K_{G}\left(S)+K_{GN}(N\right)+K_{NL}\left(q\right)$$

Through the analysis, the influence of initial prestress level S $\left(S=y_{S}S\right)$ on the following parameters is determined: Displacements q, normal forces **N**, effort of the structure $W_{max}=N_{max}/N_{Rd}$ (where: $N_{max}$ is the maximum normal force and $N_{Rd}$ is the load-bearing capacity) and stiffness of the structure, assessed by the global stiffness parameter (GSP) [20,21]:

(4) $$GSP=\frac{\;{\left[q\left(S_{min}\right)\right]}^{T}K_{S}\left(S_{min}\right)q\left(S_{min}\right)}{{\left[q\left(S_{i}\right)\right]}^{T}K_{S}\left(S_{i}\right)q\left(S_{i}\right)},$$

where $K_{S}\left(S_{min}\right)$ and $q\left(S_{min}\right)$ are a secant stiffness matrix and a design displacement vector with a minimum initial prestress level, and $K_{S}\left(S_{i}\right)$ and $q\left(S_{i}\right)$ at i-th prestress level.

### 2.2. Dynamic Analysis

The important feature of tensegrity structures is the ability to control both static and dynamic parameters. The dynamic response can be studied by modal analysis [7,14,15,16,23,24,26,27,29]. Without load, $\left(P=0\right)$ the Equation (1) is quasi-linear. Taking into account the harmonic motion $q\left(t\right)=\tilde{q}\sin\left(2\pi ft\right)$, where $\tilde{q}\left(\in {\mathbb{R}}^{m\times 1}\right)\;$ is the amplitude vector, the Equation (1) could be written as:

(5) $$\left[K_{L}+K_{G}\left(S\right)-{\left(2\pi f\right)}^{2}M\right]\tilde{q}=0.$$

The modal analysis (5) leads to the determination of the natural frequencies of vibrations $f_{i}\left(0\right)$. For a tensegrity structure characterized by mechanisms, the omission of the influence of prestress $\left(S=0\right)$ in the Equation (5) leads to zero natural frequencies. These zero values correspond to the vibrations patterns that implement the mechanisms. If the mechanism is infinitesimal, the eigenvalues of the stiffness matrix $\left[K_{L}+K_{G}\left(S\right)\right]$ are positive numbers—the prestress forces S stabilize the structure. If the eigenvalue still remains zero, then the related mechanism is not infinitesimal. In turn, if the eigenvalue are negative numbers, the structure is not stable.

Taking into account the time-independent external load P, the frequencies $f_{i}\left(P\right)$ are considered. The load is treated as the initial disturbance of the equilibrium state, i.e., as the imposition of the initial conditions. Hence, in the further part of the paper, the frequencies $f_{i}\left(P\right)$ are called free. Considering the external load, the modal analysis is non-linear. The calculations are carried out in six steps:

- Step 1—determination of the displacements from the non-linear system of equilibrium equations:

(6) $$\left[K_{L}+K_{G}\left(S)+K_{GN}(N\right)+K_{NL}\left(q\right)\right]q=P$$

Note! In this step, the structure stability should be verified. The eigenvalues of the tangent stiffness matrix $\left[K_{L}+K_{G}\left(S\right)+K_{NL}\left(q\right)\right]$ must be positive numbers.

**Figure 1:**
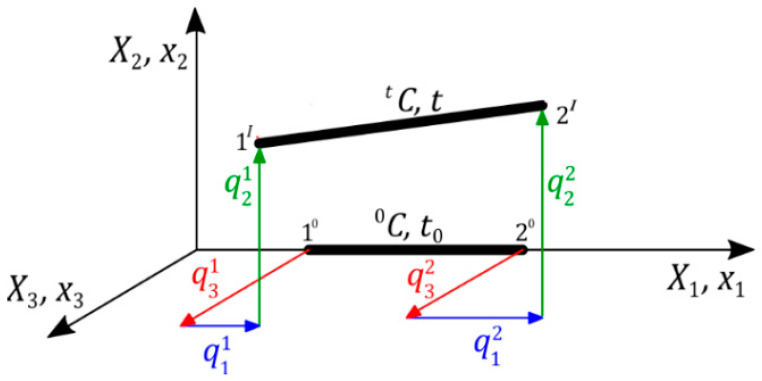
Spatial finite tensegrity element.

- Step 2—determination of deformation of elements $\varepsilon^{e}$ (a spatial finite tensegrity element in an undeformed configuration (initial) C 0 and a deformed configuration (actual) C t (Figure 1) is taken into account. In the initial configuration, the cross-sectional area and length are $A^{e}$ and $l^{e}$, respectively, whereas in the actual configuration it is $\;A_{1}^{e}$ and $l_{1}^{e}$:

(7) $$\varepsilon^{e}=\frac{1}{2}\frac{{\left(l_{1}^{e}\right)}^{2}-{\left(l^{e}\right)}^{2}}{{\left(l^{e}\right)}^{2}},$$

where

(8) $$l_{1}^{e}={\left(\Delta_{u_{2}}\right)}^{2}\sqrt{{\left(\Delta_{u_{2}}\right)}^{2}+{\left(\Delta_{u_{3}}\right)}^{2}+{\left(l^{e}+\Delta_{u_{1}}\right)}^{2}\;};{\;\Delta }_{u_{i}}=q_{i}^{2}-q_{i}^{1};\;i=1,2,3$$

- Step 3—determination of the real normal forces in elements $N^{e}$:

(9) $$N^{e}=E^{e}A^{e}\varepsilon^{e}\sqrt{1+2\varepsilon^{e}}.$$

- Step 4—determination of the geometric stiffness matrix of the structure depending on the initial prestress level $K_{G}\left(S\right)$ and normal forces $K_{GN}\left(N\right)$ caused by the external load.

Note! In this step, the prestress range should be determined. The lowest level of initial prestress $S_{min}$ must ensure the appropriate identification of the element type (cables or struts). Additionally, $S_{min}$ must provide the positive definite matrix $\left[K_{L}+K_{G}\left(S\right)+K_{GN}\left(N\right)\right]$. In turn, the maximum $S_{max}$ cannot generate the exceedance of the load-bearing capacity of elements.

- Step 5—determination of the free frequencies $f=f\left(P\right)$ from the equation:

(10) $$\left[K_{L}+K_{G}\left(S\right)+K_{GN}\left(N\right)-{\left(2\pi f\right)}^{2}M\right]\tilde{q}=0.$$

## 3. Behavior of Structures Characterized by the Self-Stress State and Infinitesimal Mechanism

**Figure 2:**
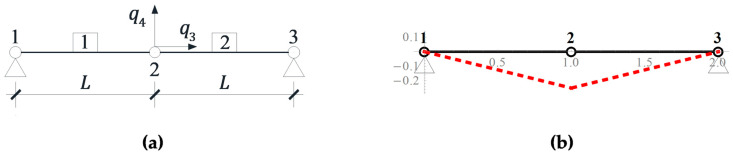
(**a**) Two-element truss and (**b**) infinitesimal mechanism.

To illustrate the behavior of the structures characterized by self-stress states and infinitesimal mechanisms, the simplest truss consisting of two elements $\left(n=2\right)$ is considered (Figure 2a) [33,34]. The elements are characterized by the Young modulus E, the cross-sectional area A and the length L. The structure is characterized by two degrees of freedom $\left(m=2\right)$ − $q={\left[\begin{array}{cc} q_{3} & q_{4} \end{array}\right]}^{T}$. The compatibility matrix takes the form:

(11) $$B=\left[\begin{array}{cc} 1 & 0\\ -1 & 0 \end{array}\right].$$

The spectral analyses (1) and (2) lead to the following eigenvalues $\begin{array}{cc} \mu =[2 & 0 \end{array}]$ and $\begin{array}{cc} \lambda =[2 & 0 \end{array}]$. The zero eigenvalues are respectively correlated to the existence of one self-stress state considered as an eigenvector $y_{2}\left(\mu_{2}=0\right)=\left[\begin{array}{cc} 1 & 1 \end{array}\right]$ (self-stress forces amount to $N^{1}=S,\;N^{2}=S$) and one mechanism considered as an eigenvector $x_{2}\left(\lambda_{2}=0\right)=\left[\begin{array}{cc} 0 & 1 \end{array}\right]$ (Figure 2b). In turn on, the eigenvalues of the problem (3) are as follows:

(12) $$\sigma_{1}=\frac{2\left(EA+S\right)}{L},\;\;\;\;\;\;\;\;\sigma_{2}=\frac{2S}{L}.$$

For real structures, the first value (12)_1_ is always a positive number, while the second value (12)_2_ depends on the value of axial force S:

- if S=0—the eigenvalue (12)_2_ is equal to zero which corresponds to the finite mechanism,

- if S>0—the eigenvalue (12)_2_ is positive and the structure is stable; it means the self-stress states stabilizes mechanism, i.e., the mechanism is infinitesimal,

- if S<0—the eigenvalue (12)_2_ is negative and the structure is instable.

This structure is not a tensegrity because it is stable only for tensile forces (there are no compressed elements—struts). Nevertheless, its behavior fully reflects the behavior of tensegrity structures and makes it possible to determine the impact of initial prestress level S on the static and dynamic parameters in explicit form. The non-linear equation of motion (3) for this truss takes the following form:

(13) $$\left\{\frac{EA}{L}\;\left[\begin{array}{cc} 2 & 0\\ 0 & 0 \end{array}\right]+\frac{S}{L}\;\left[\begin{array}{cc} 2 & 0\\ 0 & 2 \end{array}\right]+\frac{EA}{L^{3}}\;\left[\begin{array}{cc} q_{3}^{2} & q_{3}q_{4}\\ q_{3}q_{4} & q_{4}^{2} \end{array}\right]-\frac{{\left(2\pi f\right)}^{2}\rho AL}{6}\left[\begin{array}{cc} 4 & 0\\ 0 & 4 \end{array}\right]\right\}\left[\begin{array}{c} q_{3}\\ q_{4} \end{array}\right]=\left[\begin{array}{c} 0\\ P_{4} \end{array}\right]$$

where $P_{4}$ is the concentrated force applied in node 2 in the vertical direction.

In next analysis, it was assumed that the cables with length L=1 m and diameter ϕ=20 mm are made of steel with Young modulus 210 GPa and density $\rho =7860\;\mathrm{kg}/m^{3}$. In order to illustrate the influence of external loads on the behavior, four values of load $P_{4}$ are considered, i.e., $P_{4}^{1}=-0.5\;\mathrm{kN}$, $P_{4}^{2}=-1\;\mathrm{kN}$, $P_{4}^{3}=-3\;\mathrm{kN}$ and $P_{4}^{4}=-5\;\mathrm{kN}$. When applying initial prestressing forces, the load capacity $N_{Rd}=110.2\;\mathrm{kN}$ [35] did not exceed 85% ($S_{max}=70\;\mathrm{kN})$.

### 3.1. Static Analysis

In the static analysis, due to the symmetry of the structure and load, the displacement *q_3_* is zero, and the Equation (13) takes the form of a static equilibrium:

(14) $$\left(\frac{2S}{L}+\frac{EA}{L^{3}}q_{4}^{2}\right)q_{4}=P_{4}.$$

The application of non-linear theory (III order theory) takes into account the stiffening of the structure under the influence of external load, which is responsible for the displacements consistent with the infinitesimal mechanism $\left(\frac{EA}{L^{3}}q_{4}^{3}\right)$. If this influence is neglected (II order theory), the solution of Equation (14) leads to the following relationship:

(15) $$q_{4}=\frac{P_{4}L}{2S}.$$

**Figure 3:**
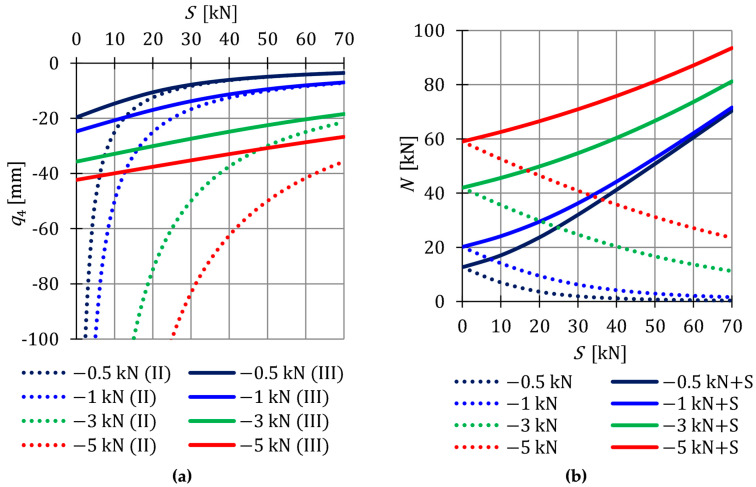
Impact of the initial prestress level *S* on the: (**a**) Displacement $q_{4}$ and (**b**) axial force N.

The absence of initial prestress (S=0) caused the displacement (15) to increase to infinity. The impact of the initial prestress level *S* on the displacement $q_{4}$ is shown in Figure 3a.

The stiffness of the considered structure is not only conditioned on the geometry and material characteristics, but also on the level of initial prestress S, which stabilizes the infinitesimal mechanisms, and on the external load $P_{4}^{i}$. With the increase of prestressing forces, the differences between the calculations made according to the second and third order theory are decreasing. The influence of non-linearity is most significant at low values of initial prestress forces. With lower values of the load, the initial prestress has a higher impact on the total rigidity of the structure—the differences between the displacements obtained using the second and third order theory at $P_{4}^{1}=-0.5\;\mathrm{kN}$ are smaller than at $P_{4}^{4}=-5\;\mathrm{kN}$. The external load prestresses the structure—additional tensile forces are generated in the cables. However, after introducing the initial prestress, the normal forces from the external load successively decrease, and thus its influence on the displacement decreases. Figure 3b shows the change in the value of normal forces arising from loads $\left(P\right)$ and normal forces generated jointly by the load and prestress forces $\left(P+S\right)$.

In the presented structure, there is only one non-zero displacement, so the assessment of the behavior of this displacement (local assessment) is also an assessment of the behavior of the entire structure (global assessment). In the case of structures with many degrees of freedom, it is not possible, to trace all displacements for objective reasons. Therefore, the authors propose a parameter that helps to assess the influence of the self-stress state on the total rigidity of the structure at a given load. The literature on tensegrity structures does not contain any parameter characterizing the change in rigidity. In the paper, the global stiffness parameter (GSP) (6) is used. In the case of the analyzed structure, the nature of changes in the GSP can be expressed explicitly:

(16) $$GSP=\frac{q_{4}\left(0\right)}{q_{4}\left(S_{i}\right)}$$

**Figure 4:**
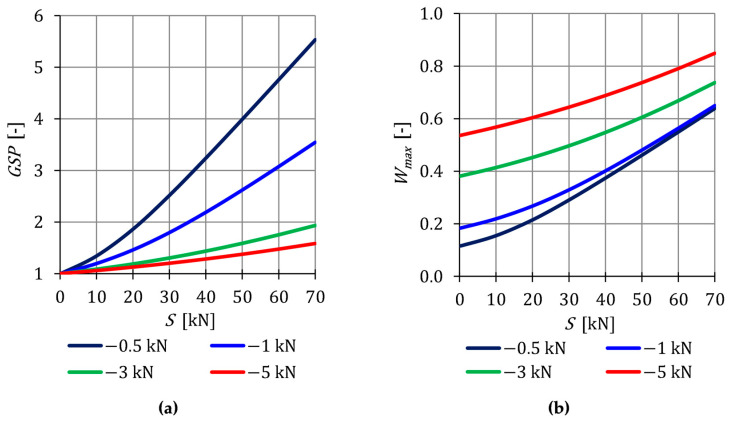
Impact of the initial prestress level *S* on the: (**a**) Global stiffness parameter GSP and (**b**) effort of structure $W_{\max}$.

The parameter (16) is presented in Figure 4a. At the maximum of initial prestress for $P_{4}^{1}$, GSP is 3.5 times higher than for $P_{4}^{4}$. This confirms the previous conclusions that, with lower external load, the initial prestress forces have a higher impact on the overall stiffness of the structure. Additionally, due to the effect of the initial prestress forces on the normal forces N, the effort of structure $W_{\max}$, depending on the level of initial prestress, is also monitored (Figure 4b). In the case of the effort of the structure, as in the case of the stiffness, the influence of initial prestress decreases as the load increases.

### 3.2. Dynamic Analysis

In the dynamic analysis, Equation (13) for the two-element truss takes the following form:

(17) $$\left\{\frac{EA}{L}\;\left[\begin{array}{cc} 2 & 0\\ 0 & 0 \end{array}\right]+\frac{S}{L}\;\left[\begin{array}{cc} 2 & 0\\ 0 & 2 \end{array}\right]-\frac{{\left(2\pi f\right)}^{2}\rho AL}{6}\left[\begin{array}{cc} 4 & 0\\ 0 & 4 \end{array}\right]\right\}\left[\begin{array}{c} q_{3}\\ q_{4} \end{array}\right]=\left[\begin{array}{c} 0\\ 0 \end{array}\right]$$

The non-trivial solution of Equation (17) leads to the determination of the natural frequencies:

(18) $$f_{1}=\sqrt{\frac{3S}{2\pi \rho AL^{2}}},\;\;\;\;\;\;\;\;\;f_{2}=\sqrt{\frac{3\left(EA+S\right)}{2\pi \rho AL^{2}}},$$

and corresponding to them the vibration modes:

(19) $$\tilde{q}(f_{1})=\left[\begin{array}{c} 0\\ 1 \end{array}\right],\;\;\tilde{q}(f_{2})=\left[\begin{array}{c} 1\\ 0 \end{array}\right].$$

The most dependent on the self-stress state is the first frequency (18)_1_:

- if S=0 —the frequency (18)_1_ is equal to zero which corresponds to the mechanism described by the vibration mode (19)_1_ (Figure 4a),

- if S>0 —the frequency (18)_1_ is positive and increases proportionally to the square root of the prestressing amplitude,

- if S<0 —the frequency (18)_1_ is an imaginary number and the structure is instable.

**Figure 5:**
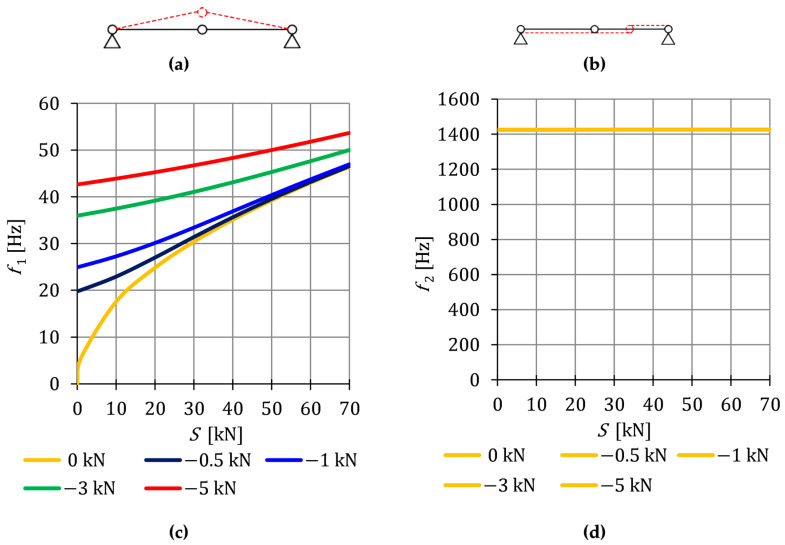
Vibration modes: (**a**) The first mode (19)_1_ and (**b**) the second mode (19)_2_; Impact of the initial prestress level *S* on the: (**c**) First frequency (18)_1_ and (**d**) second frequency (18)_2_.

In the case of the second frequency (18)_2_, the influence of initial prestress is negligible, because under the condition of the bearing capacity, the values of prestressing forces S are much lower than the longitudinal stiffness (S ≪ EA). The second form of vibrations (Figure 5b) is described by the vector (19)_2_.

The impact of initial prestress S on the natural frequency $f_{i}\left(0\right)$ is showed in Figure 5. The value of the first frequency varies from $f_{1}\left(0\right)=0$ to $f_{1}\left(0\right)=44.6\;\mathrm{Hz}$ (Figure 5c), while the second frequency is practically insensitive to the change—at the prestress level at S=0 it is $f_{2}\left(0\right)=1424.9\;\mathrm{Hz}$ and at $S_{max}=70\;\mathrm{kN}$—1425.6 Hz (Figure 5c).

Additionally, the free frequencies $f_{i}\left(P\right)$ are calculated on the basis of the Equation (10). In the case of the first frequency (Figure 5c), the external load prestresses the structure —additional tensile forces are generated in the cables and the initial dynamic response (at S=0) corresponds to the values of the natural frequency at the following force levels: $N\left(P_{4}^{1}=-0.5\;\mathrm{kN}\right)=12.72\;\mathrm{kN}$, $N\left(P_{4}^{2}=-1\;\mathrm{kN}\right)=20.20\;\mathrm{kN},$ $N\left(P_{4}^{3}=-3\;\mathrm{kN}\right)=42.11\;\mathrm{kN}$ and $N\left(P_{4}^{4}=-5\;\mathrm{kN}\right)=59.12\;\mathrm{kN}$. The second free frequency does not depend on the initial prestress and is equal to the natural frequency—$f_{2}\left(0\right)=f_{2}\left(P\right)$.

The conducted analysis showed that the influence of the self-stress state on the first vibration frequency diminishes with the rise in the load value. The second vibration frequency is not correlated to both the change in the level of prestress and the impact of external loads.

## 4. Tensegrity Structures

The paper presents static and dynamic parametric analyzes of tensegrity structures built with the use of modified Simplex and Quartex modules. The dimensions of the considered single module allow it to fit into a unit cube. By inscribing the upper surface of the modules into the lower one, it is possible to easily combine single units into multi-module structures. A linear connection is considered with different ways of connecting modules and different support conditions.

Firstly, the immanent features are determined (qualitative analysis). Next, the influence of initial prestress level on static and dynamic parameters of the structures is considered (quantitative analysis). The time-independent external loads are taken into account. To perform the calculations, a program using a geometrically non-linear model was written in the Mathematica environment.

### 4.1. Qualitative Analysis

**Figure 6:**
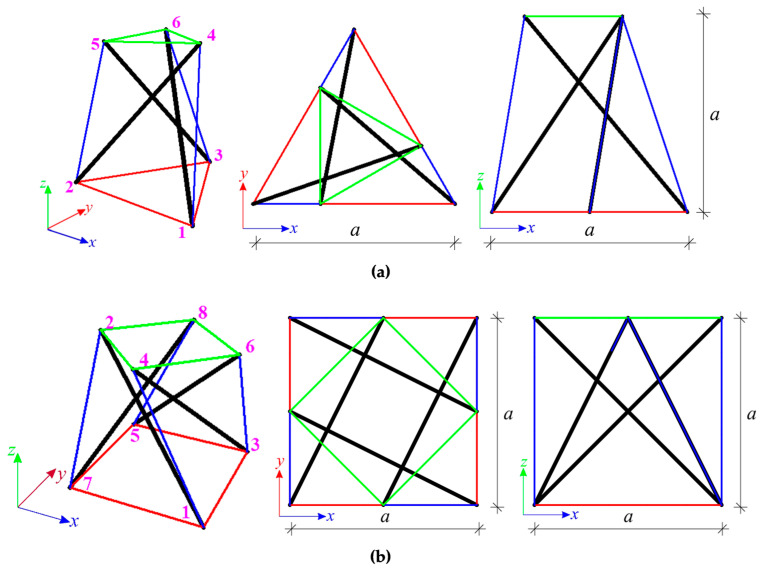
Simple modified modules: (**a**) Simplex and (**b**) Quartex.

The qualitative analysis leads to the identification of immanent features. Thanks to this, the geometric and mechanical properties do not influence the unique properties of tensegrity, and all constants were assumed as unitary so the elasticity matrix is an identity matrix E=I. The first considered structures are the single modified Simplex (Figure 6a) and Quartex (Figure 6b) modules. Two support conditions variants are considered. In the first case, the number of elements and the number of degrees of freedom are equal—the models with six (for Simplex—S1-1) and eight (for Quartex—Q1-1) bonds are considered. In the second variant, all degrees of freedom of the bottom nodes are taken away—the models with nine (S1-2) and twelve (Q1-2) bonds are considered, respectively. The results of the qualitative analysis for the single modules are showed in Table 1.

**Figure 7:**
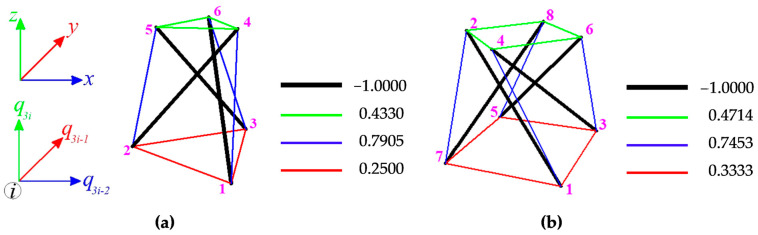
Values of normalized self-stress states $y_{S}$: (**a**) Simplex and (**b**) Quartex.

In the first case of support, the modules are characterized by one self-stress and one mechanism. In Figure 7, the values of normalized self-stress forces (force in struts is equal to −1) are shown. The cables are marked in red (bottom), green (top) and blue (diagonal), whereas the struts—in black. The different colors of cables correspond to the different values of the self-stress state. In the second support variant, one mechanism is identified for both modules, but the number of self-stress states is different. For Simplex module four self-stress states are identified, whereas for the Quartex module it is five. The identified self-stress states do not stabilize the modules. In such case, a superposition of the all self-stress states is required. The superposition leads to the prestressing forces obtained for the first support variant.

**Figure 8:**
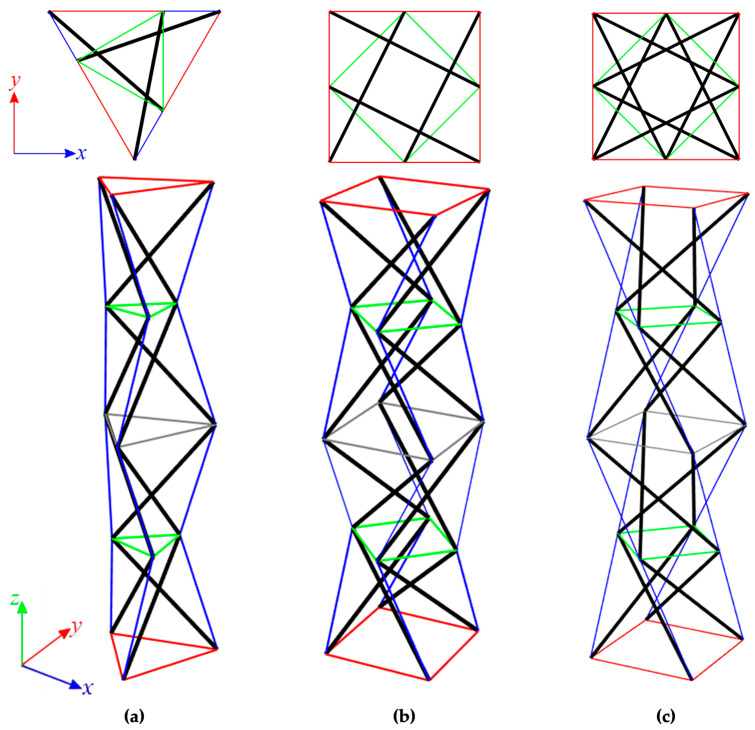
Linear four-module models: (**a**) S4, (**b**) Q4-connection A, and (**c**) Q4-connection B.

The structures built with linearly connected modules (towers) are considered as next. The units are connected node-to-node. The Simplex modules could be connected only in one way (Figure 8a), whereas the Quartex modules—in two ways (Figure 8b,c). In the case of the connection A, the struts overlap in a plan view, while in the connection B, they form a star. The structures built with *n* (*n* = 2, 3, 4, 5, 6) modules are considered. The results of the qualitative analysis for tensegrity towers built with Simplex (S*n*) and Quartex (Q*n*) modules are shown in Table 2 and Table 3, respectively.

Regardless of the type of modules and the way they are connected, the structures behave the same. Only support conditions affect the result of the analysis. In the first case, i.e., Simplex towers with six bonds and Quartex ones with eight bonds, the number of self-stress states and mechanisms are equal the number of modules. In the second case (models with nine and twelve bonds), the number of identified mechanisms does not change, but the number of self-stress states increases by three for Simplex models and by four for Quartex models. None of the identified self-stress states stabilize the towers. Only the superposition of self-stress states from the single modules ensures the stability of structures.

### 4.2. Quantitative Analysis

Through the quantitative analysis, the influence of the initial prestress level S $\left(S=y_{S}S\right)$ on the static and dynamic parameters is considered. It was assumed that the parameter a presented in Figure 5 is equal a=1 m and the structures are made of steel with density $\rho =7860\;\mathrm{kg}/m^{3}$. The cables with diameter ϕ=20 mm are made of steel S460N with the load-bearing capacity $N_{Rd}=110.2\;\mathrm{kN}$ [35]. The type A cables with Young modulus E=210 GPa are used. The struts are made of a hot-finished circular hollow section with diameter ϕ=76.1 mm and thickness t=2.9 mm (steel S355J2) with the Young modulus E=210 GPa and the load-bearing capacity $N_{Rd}=203.5\;\mathrm{kN}$ [36] for the Simplex module and $N_{Rd}=193.9\;\mathrm{kN}$ for the Quartex module [36]. The calculations were made using a geometrically non-linear model implemented in a proprietary program written in the Mathematica environment. For the single modules, the minimum prestress value is assumed as $S_{min}=0\;\mathrm{kN}$, whereas for structures—$S_{min}=5\;\mathrm{kN}$. The maximum value is assumed as $S_{max}=110\;\mathrm{kN}$.

#### 4.2.1. Static Analysis

**Figure 9:**
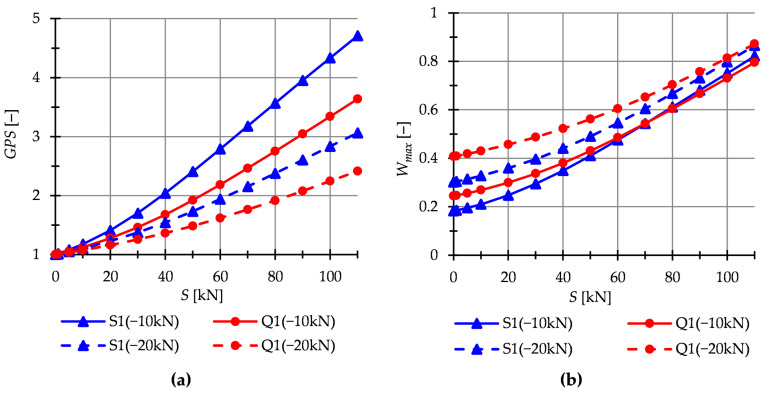
Single modules. Impact of the initial prestress level *S* on the: (**a**) Global stiffness parameter GSP and (**b**) effort of the structure (cables) $W_{\max}$.

**Figure 10:**
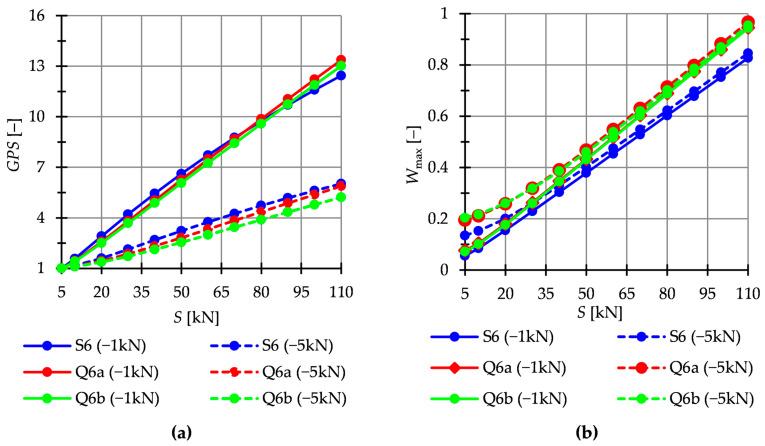
Structures build with six modules. Impact of the initial prestress level *S* on the: (**a**) Global stiffness parameter GSP and (**b**) effort of structure $W_{\max}$.

In this paper, the static analysis includes the determination of the influence of the level of initial prestress S on the global stiffness parameter GSP and the effort of structure $W_{\max}$. The structures are loaded with a *z*-direction force applied to one top node. Two load variants are considered. For the single modules, it is $P_{z}^{1}=-10\;\mathrm{kN}$ and $P_{z}^{2}=-20\;\mathrm{kN}$ (Figure 9), whereas for the six-modules structures—$P_{z}^{1}=-1\;\mathrm{kN}$ and $P_{z}^{2}=-5\;\mathrm{kN}$ (Figure 10).

The Simplex module is more sensitive to changes in the level of initial prestress. At the maximum level of initial prestress, regardless of the load variant, the GSP for Simplex is 1.3 times higher than for Quartex. In the case of multi-module structures (Figure 10), the behavior is comparable.

#### 4.2.2. Dynamic Analysis

**Figure 11:**
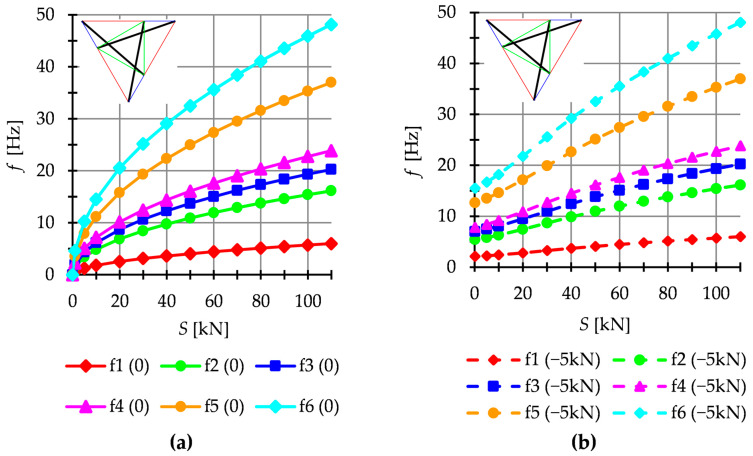
Simplex structure S6: (**a**) Natural frequencies $fi\;\left(0\right)$ and (**b**) free frequencies $fi\;\left(P\right)$.

**Figure 12:**
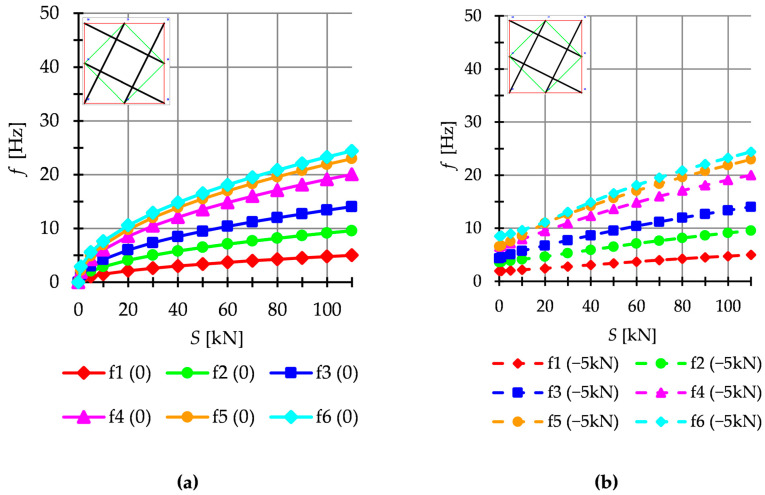
Quartex structure Q6a: (**a**) Natural frequencies $fi\;\left(0\right)$ and (**b**) free frequencies $fi\;\left(P\right)$.

**Figure 13:**
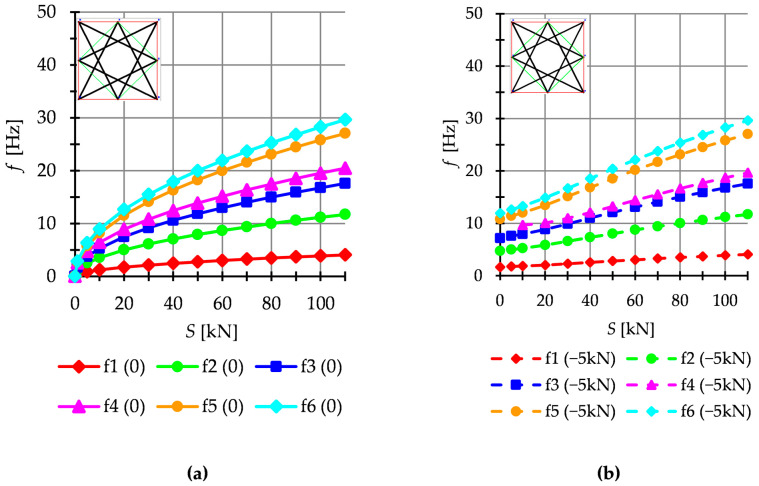
Quartex structure Q6b: (**a**) Natural frequencies $fi\;\left(0\right)$ and (**b**) free frequencies $fi\;\left(P\right)$.

The dynamic analysis included calculations of the natural vibrations $f\left(0\right)$ and of the free vibrations of structures loaded with time-independent force $f\left(P\right)$. The structures are loaded with a *z*-direction force applied to one top node. Only one case of load is taken into account, i.e., P=−5 kN. The influence of initial prestress S on the dynamic response of structures is considered. For example, the dynamic behavior of the six-modules structure is presented. The structures built with the Simplex modules are presented in Figure 11, while structures built with the Quartex modules are showed in Figure 12 (Q6a) and Figure 13 (Q6b). The number of the natural frequencies, which are zero in the case of S=0, is equal to the number of infinitesimal mechanisms. In all cases, it is six frequencies. These frequencies increase when the initial prestress is applied. Higher frequencies are more susceptible to the change in prestressing. Generally, the natural frequencies of the structure built with the Simplex modules (S6) are higher in the structure built with the Quartex (Q6a, Q6b) modules. The value of the six frequency for S6 varies from 0 to 48.14 Hz, for Q6a—from 0 to 24.40 Hz and for Q6b—from 0 to 29.63 Hz. The obtained results confirm that the way of connecting the Quartex modules is important. For the structure with star-forming struts (Q6b), the six frequencies for $S_{\max}$ is 1.3 times higher than for the structure with struts overlapping in a plan view (Q6a). The free frequencies $fi\left(P\right)$ are calculated from the Equation (10). The external load prestresses the structures, and the initial dynamic responses at S=0 are not zero (Figure 11b, Figure 12b and Figure 13b). As the prestress increases, the free frequencies become equal the natural frequencies.

**Figure 14:**
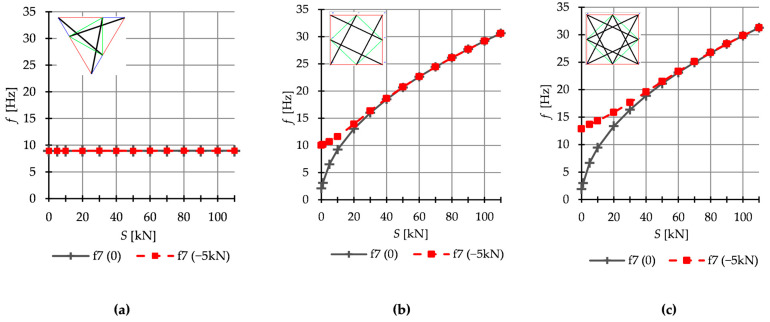
Seventh natural $f7\;\left(0\right)\;$ and free $f7\;\left(P\right)$ frequencies for: (**a**) S6, (**b**) Q6a, and (**c**) Q6b.

Theoretically, it is well known that the number of natural frequencies, depending on the prestressing, is equal to the number of infinitesimal mechanisms. However, in the case of some analyzed structures it is different. Only structures built with Simplex modules behave according to this rule. In Figure 14, the seventh natural and free frequency for structures built with six modules is showed. In the case of the Simplex structures (Figure 14a), the natural and free are the same $f7\left(0\right)=f7\left(P\right)$ and do not depend on the initial prestress. However, for the Quartex structures (Figure 14b,c), the seventh frequency is additionally dependent on initial prestress. In the absence of initial prestress $\left(S=0\right)$, the natural frequency is not zero and their values vary with the change of prestress. The free seventh frequency behaves the same. The structures built with four and five Quartex modules behave similarly. Table 4 shows all values of natural frequencies sensitive to the change in the level of self-stress (white cells) and the following value of the natural frequency independent of the prestress level (blue cells). Yellow cells indicate the abnormal behavior on the structure—these values should theoretically also remain independent of the prestress level, but they are not.

**Figure 15:**
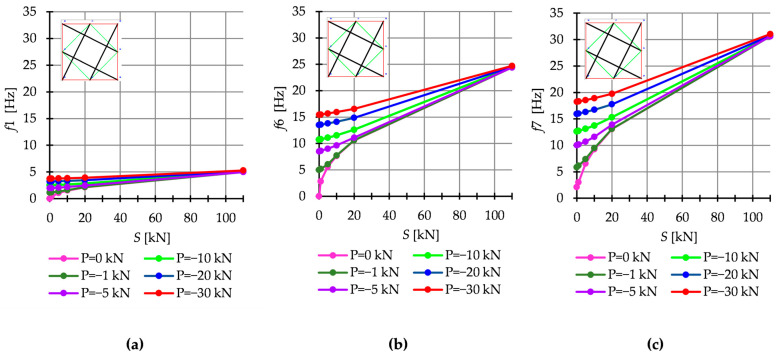
Natural and free frequency for Q6a: (**a**) *f*1, (**b**) *f*6, and (**c**) *f*7.

The dynamic response of tensegrity structures is also affected by external loads. To illustrate this fact, as an example, the free frequency for the Q6a model for different values of the external load are shown in Figure 15. Regardless of the number of frequencies, the impact of loads is greater at a lower level of initial prestress and as the prestress increases, the free frequencies become equal the natural frequencies. Additionally, for the level of self-stress higher than S>20 kN, the relationship frequency-prestress becomes linear.

## 5. Conclusions

In this paper, the static and dynamic behavior of tensegrity structures is explored. First, to illustrate the behavior of the structures characterized by self-stress states and infinitesimal mechanisms, the simplest truss, consisting of two elements, is considered. For such a simple structure, it is possible to obtain the static and dynamic parameters in an explicit form. This approach makes it easier to understand behavior of tensegrity structures. Next, the structures built of linearly connected the most popular tensegrity modules (modified Simplex and Quartex modules) are considered. Two ways of connecting the Quartex modules are considered, i.e., connection A—the struts overlap in a plan view, and connection B—the struts form a star. Additionally, different support conditions are analyzed. Particularly, the impact of initial prestress on the static and dynamic parameters is analyzed.

In the case of the qualitative analysis, which leads to the identification of immanent features, regardless of the type of modules and the way they are connected, the structures behave the same. Only the support conditions affect the result of the analysis. In contrast, for the quantitative static and dynamic analysis, the behavior of structures depends on the type of modules and the way they are connected.

In the dynamic analysis of tensegrity structures, it is well known that the number of prestress-dependent natural frequencies is equal to the number of infinitesimal mechanisms. With no prestress, these frequencies are zero, and the correlated forms of vibrations implement the mechanisms. After applying the self-stress state, the frequencies increase in proportion to the square root of that state. The sensitivity of these natural frequencies to the self-stress state is so great that the change in the level of prestress can be auspiciously used to control the dynamic properties of the structure. Other frequencies theoretically should be practically insensitive to self-stress changes. If several mechanisms are identified, the higher frequencies are more susceptible to the initial prestress changes.

Considering the examples presented in this paper, the highest frequencies were obtained for the structures built with the Simplex module, followed by structures built with the modified Quartex with the struts forming a star, while the lowest frequencies were obtained for the structures built with the modified Quartex with the overlapping struts. In addition, the Simplex towers behave typically, whereas both Quartex towers exhibit an abnormality in the dynamic analysis. Theoretically, as mentioned before, the number of natural frequencies, depending on the prestressing, is equal to the number of infinitesimal mechanisms. However, in the case of some analyzed structures it is different. Only structures built with Simplex modules behave according to this rule. However, for the Quartex structures, there is an additional frequency dependent on the initial prestress. In the absence of initial prestress $\left(S=0\right)$, the natural frequency is not zero and its value varies with the change of prestress. The additional free frequency behaves the same. The considerations contained in this paper indicate the unusual behavior of tensegrity structures. The obtained results are important for the dynamic stability analysis of the behavior of tensegrity structures under the periodic loads, which will be the subject of the second part of the paper. The dynamic stability analysis cannot be carried out without the analysis presented in this paper.

## Figures and Tables

**Table 1:** Results of the qualitative analysis for single modules.

No. of:	S1		Q1	
elements	12		16	
nodes	6		8	
bonds	S1-1	S1-2	Q1-1	Q1-2
	6	9	8	12
degrees of freedom	12	9	16	12
mechanisms	1	1	1	1
self-stress states	1	4	1	5

**Table 2:** Results of the qualitative analysis for linear models built with Simplex modules.

No. of:	S2		S3		S4		S5		S6	
elements	21		30		39		48		57	
nodes	9		12		15		18		21	
bonds	S2-1	S2-2	S3-1	S3-2	S4-1	S4-2	S5-1	S5-2	S6-1	S6-2
	6	9	6	9	6	9	6	9	6	9
degrees of freedom	21	18	30	27	39	36	48	45	57	54
mechanisms	2	2	3	3	4	4	5	5	6	6
self-stress states	2	5	3	6	4	7	5	8	6	9

**Table 3:** Results of the qualitative analysis for linear models built with Quartex modules.

No. of:	Q2		Q3		Q4		Q5		Q6	
elements	28		40		52		64		76	
nodes	12		16		20		24		28	
bonds	Q2-1	Q2-2	Q3-1	Q3-2	Q4-1	Q4-2	Q5-1	Q5-2	Q6-1	Q6-2
	8	12	8	12	8	12	8	12	8	12
degrees of freedom	28	24	40	36	52	48	64	60	76	72
mechanisms	2	2	3	3	4	4	5	5	6	6
self-stress states	2	6	3	7	4	8	5	9	6	10

**Table 4:** Natural frequencies.

	f1		f2		f3		f4		f5		f6		f7		f8	
	0	$S_{max}$	0	$S_{max}$	0	$S_{max}$	0	$S_{max}$	0	$S_{max}$	0	$S_{max}$	0	$S_{max}$	0	$S_{max}$
S1	0	37.8	152.6	152.8												
Q1a		24.6	200.8	201.2												
Q1b		24.6	200.8	201.2												
S2	0	14.5	0	34.7	67.9	68.0										
Q2a		11.6		21.2	62.3	62.4										
Q2b		11.2		29.2	64.1	64.2										
S3	0	11.0	0	29.9	0	39.8	31.4	31.4								
Q3a		8.9		15.7		27.2	28.5	38.0	29.4	29.5						
Q3b		8.1		25.7		30.1	29.8	38.9	31.3	31.4						
S4	0	8.1	0	20.3	0	25.2	0	39.6	17.2	17.2						
Q4a		6.7		12.3		17.0		23.0	10.7	25.8	16.5	16.6				
Q4b		6.0		16.2		23.2		27.6	11.2	30.9	17.8	17.8				
S5	0	7.1	0	18.5	0	22.7	0	38.0	0	48.8	12.6	12.7				
Q5a		5.9		11.0		16.2		24.4		29.2	5.2	32.2	11.6	11.7		
Q5b		4.9		14.0		25.1		25.9		28.5	4.8	31.0	11.7	11.8		
S6	0	6.0	0	16.2	0	20.3	0	23.9	0	37.0	0	48.1	9.0	9.0		
Q6a		5.0		9.6		14.0		20.1		23.0		24.4	2.1	30.6	8.4	8.4
Q6b		4.1		11.7		17.6		20.4		27.0		29.6	1.9	31.3	8.1	8.1

## Data Availability

The data presented in this study are available within the text of the papper.

## References

[1] Lee S., Lieu Q.X., Vo T.P., Lee J. (2022). Deep Neural Networks for Form-Finding of Tensegrity Structures. Mathematics.

[2] Wang Y., Xian X., Luo Y. (2021). Form-Finding of Tensegrity Structures via Rank Minimization of Force Density Matrix. Eng. Struct..

[3] Zhang P., Zhou J., Chen J. (2021). Form-Finding of Complex Tensegrity Structures Using Constrained Optimization Method. Compos. Struct..

[4] Song K., Scarpa F., Schenk M. (2022). Form-Finding of Tessellated Tensegrity Structures. Eng. Struct..

[5] Sultan C., Skelton R. (2002). Linear Dynamics of Tensegrity Structures. Eng. Struct..

[6] Masic M., Skelton R.E. (2006). Selection of Prestress for Optimal Dynamic/Control Performance of Tensegrity Structures. Int. J. Solids Struct..

[7] Ali N.B.H., Rhode-Barbarigos L., Albi A.A.P., Smith I.F. (2010). Design Optimization and Dynamic Analysis of a Tensegrity-Based Footbridge. Eng. Struct..

[8] Lee S., Lee J. (2014). Optimum Self-Stress Design of Cable–Strut Structures Using Frequency Constraints. Int. J. Mech. Sci..

[9] Caluwaerts K., Carbajal J.P. (2015). Energy Conserving Constant Shape Optimization of Tensegrity Structures. Int. J. Solids Struct..

[10] Skelton R.E., Pinaud J.P., Mingori D.L. (2001). Dynamics of the Shell Class of Tensegrity Structures. J. Frankl. Inst..

[11] Skelton R., Ulbrich H., Günthner W. (2005). Dynamics and Control of Tensegrity Systems. Proceedings of the IUTAM Symposium on Vibration Control of Nonlinear Mechanisms and Structures.

[12] Oliveto N.D., Sivaselvan M.V. (2011). Dynamic Analysis of Tensegrity Structures Using a Complementarity Framework. Comput. Struct..

[13] Fraternali F., Senatore L., Daraio C. (2012). Solitary Waves on Tensegrity Lattices. J. Mech. Phys. Solids.

[14] Faroughi S., Lee J. (2014). Geometrical Nonlinear Analysis of Tensegrity Based on a Co-Rotational Method. Adv. Struct. Eng..

[15] Faroughi S., Lee J. (2015). Analysis of Tensegrity Structures Subject to Dynamic Loading Using a Newmark Approach. J. Build. Eng..

[16] Ashwear N., Tamadapu G., Eriksson A. (2016). Optimization of Modular Tensegrity Structures for High Stiffness and Frequency Separation Requirements. Int. J. Solids Struct..

[17] Kan Z., Peng H., Chen B., Zhong W. (2018). Nonlinear Dynamic and Deployment Analysis of Clustered Tensegrity Structures Using a Positional Formulation FEM. Compos. Struct..

[18] Domer B., Fest E., Lalit V., Smith I.F.C. (2003). Combining Dynamic Relaxation Method with Artificial Neural Networks to Enhance Simulation of Tensegrity Structures. J. Struct. Eng..

[19] Gilewski W., Kłosowska J., Obara P. (2017). The influence of self-stress on the behavior of tensegrity-like real structure. MATEC Web Conf..

[20] Gilewski W., Obara P., Kłosowska J. (2018). Self-stress control of real civil engineering tensegrity structures. AIP Conf. Proc..

[21] Obara P., Tomasik J. (2021). Parametric Analysis of Tensegrity Plate-Like Structures: Part 2—Quantitative Analysis. Appl. Sci..

[22] Obara P., Tomasik J. (2021). Active Control of Stiffness of Tensegrity Plate-like Structures Built with Simplex Modules. Materials.

[23] Oppenheim I.J., Williams W.O. (2001). Vibration of an Elastic Tensegrity Structure. Eur. J. Mech. A Solids.

[24] Murakami H., Nishimura Y. (2001). Static and Dynamic Characterization of Regular Truncated Icosahedral and Dodecahedral Tensegrity Modules. Int. J. Solids Struct..

[25] Chen Y., Feng J. (2012). Initial Prestress Distribution and Natural Vibration Analysis of Tensegrity Structures Based on Group Theory. Int. J. Struct. Stab..

[26] Murakami H., Nishimura Y. (2001). Static and Dynamic Characterization of Some Tensegrity Modules. J. Appl. Mech.

[27] Bel Hadj Ali N., Smith I.F.C. (2010). Dynamic Behavior and Vibration Control of a Tensegrity Structure. Int. J. Solids Struct..

[28] Bolotin V.V. (1956). Dinamiczeskaja Ustojcziwost Uprugich Sistiem.

[29] Gilewski W., Kłosowska J., Obara P. (2019). Parametric analysis of some tensegrity structures. MATEC Web Conf..

[30] Murakami H. (2001). Static and Dynamic Analyses of Tensegrity Structures. Part 1. Nonlinear Equations of Motion. Int. J. Solids Struct..

[31] Bathe K.J. (1996). Finite Element Procedures in Engineering Analysis.

[32] Zienkiewicz O.C., Taylor R.L., Zhu J.Z. (2013). The Finite Element Method: Its Basis and Fundamentals.

[33] Obara P., Tomasik J. (2020). Parametric Analysis of Tensegrity Plate-Like Structures: Part 1—Qualitative Analysis. Appl. Sci..

[34] Motro R. (1992). Tensegrity Systems: The State of the Art. Int. J. Solids Struct..

[35] (2006). Eurocode 3: Design of Steel Structures—Part 1–11: Design of Structures with Tension Components.

[36] (2005). Eurocode 3: Design of Steel Structures—Part 1–1: General Rules and Rules for Buildings.

